# A Review of the Properties of Anthocyanins and Their Influence on Factors Affecting Cardiometabolic and Cognitive Health

**DOI:** 10.3390/nu13082831

**Published:** 2021-08-18

**Authors:** Philipp Ockermann, Laura Headley, Rosario Lizio, Jan Hansmann

**Affiliations:** 1 Institute for Tissue Engineering and Regenerative Medicine, University Hospital Wuerzburg, Roentgenring 11, 97070 Wuerzburg, Germany; jan.hansmann@uni-wuerzburg.de; 2 Evonik Operations GmbH, 64293 Darmstadt, Germany; laura.headley@evonik.com; 3 Evonik Operations GmbH, 63457 Hanau, Germany; rosario.lizio@evonik.com

**Keywords:** anthocyanins, antioxidative, blood pressure, hyperlipidemia, diabetes, inflammation

## Abstract

The incidence of cardiovascular and metabolic diseases has increased over the last decades and is an important cause of death worldwide. An upcoming ingredient on the nutraceutical market are anthocyanins, a flavonoid subgroup, abundant mostly in berries and fruits. Epidemiological studies have suggested an association between anthocyanin intake and improved cardiovascular risk, type 2 diabetes and myocardial infarct. Clinical studies using anthocyanins have shown a significant decrease in inflammation markers and oxidative stress, a beneficial effect on vascular function and hyperlipidemia by decreasing low-density lipoprotein and increasing high-density lipoprotein. They have also shown a potential effect on glucose homeostasis and cognitive decline. This review summarizes the effects of anthocyanins in in-vitro, animal and human studies to give an overview of their application in medical prevention or as a dietary supplement.

## 1. Introduction

The worldwide trend of “living healthy” has increased over the last few years with dietary supplements playing a more and more important role in widening access to phytochemicals that otherwise may not be readily available in daily dietary patterns. The market of nutraceutical phytochemicals had an estimated revenue of 16.1 billion $ in North America and Europe in 2018 and was estimated to increase to a revenue of 24.6 billion $ in 2023 [1]. As a subgroup of this, flavonoids have experienced considerable growth in demand, with a worldwide total revenue of 939.7 million $ in 2017 and a projected increase to a revenue of 1216.5 million $ in 2022 [2]. Anthocyanins are a flavonoid subgroup that occur in many plants, but are mostly known for their abundance in berries, where they contribute to their coloring and help protect against environmental stressors [3]. A flavonoid-rich diet is generally correlated with a lower risk of myocardial infarction, cardiovascular disease (CVD) mortality and coronary heart disease (CHD) [4,5,6]. 

There is increasing expansion of the availability and diversity of berry-derived dietary supplements available to the consumer, often containing extracts from a range of anthocyanin-rich berries, such as bilberry (*Vaccinium myrtillus*), blueberry (species such as *Vaccinium angustifolium* or *Vaccinium corybosum*), and maqui berry (*Aristotelia chilensis*), as well as tart cherry (*Prunus cerasus*) and blackcurrant (*Ribes nigrum*).

At present, there are no authorized health claims from the US Food and Drug Administration (FDA) or the European Food Safety Authority (EFSA) referring to anthocyanins. However, vendors continue to attempt to convey the apparent health benefits of anthocyanin supplementation to the consumer via their interpretation of the literature base investigating the bioactivity of anthocyanins, describing effects on the function of the eyes, vasculature, lipid profile, liver, anti-oxidant status and anti-inflammatory effects. 

Although there is no approved health claim yet, numerous studies have examined the effect of anthocyanins both in vitro and in vivo. The most promising effect seems to be regarding cardiovascular disease, which has grown to be more important over the last decades as a target for public health intervention. From 2007 to 2017, the global prevalence for CVD has increased by 28.5% and the deaths attributed to CVD have increased by 21.1% [7]. Therefore, there is high interest in the effects of anthocyanin supplementation, not only on the disease itself, but also as a possible preventative health intervention. Due to the variety of studies conducted involving anthocyanins, it is timely to now review the most promising recorded effects in in vitro and in vivo studies from animals and humans to give an overview on the current scientific state with emphasis on cardiovascular disease-related parameters. 

In order to understand the scope of influence of anthocyanins, this narrative review focusses on the absorption and metabolization of anthocyanins, progressing on to examining both their anti-inflammatory and anti-oxidative activity. How anthocyanins may influence key risk factors for CVD, particularly the reported down-stream effects on general vascular function (e.g., blood pressure), hyperlipidemia and glucose homeostasis, will also be examined. In addition, neuroinflammation has similar underlying mechanisms to CVD and is considered to be a contributor to neurodegenerative diseases such as Alzheimer’s disease [8,9]. Due to this connection, there are a number of pertinent studies reporting the effect of anthocyanins on cognition and neurodegenerative diseases that warrant describing and assimilating.

## 2. Overview

**Figure 1:**
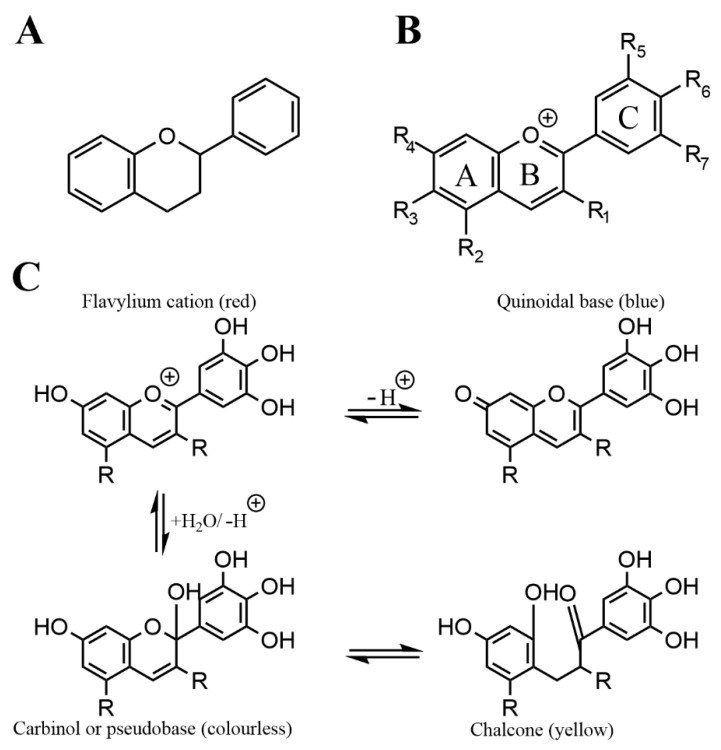
Chemical structure of flavan (**A**), the general anthocyanin structure (**B**) and the structure and color change of anthocyanin with pH in aqueous solution (**C**).

Anthocyanins are a subgroup of flavonoids, which are a prevalent and numerous class of secondary plant metabolites, that all share the C_6_-C_3_-C_6_ flavan or 2-phenyl-benzodihydorpyrane skeleton (Figure 1A). In plants, they are used for pigmentation, UV-filtration, protection from reactive oxygen species or nitrogen fixation. Other subgroups, based on chemical structure, are anthoxanthins, flavanones, flavanolols, or flavans.

To date, there are more than 500 different anthocyanins known, which makes them the biggest group of water-soluble plant pigments [10]. Their name derives from the greek *anthos* (flower) and *kyanous* (dark blue). They are based on the flavylium cation, which makes them electrically charged at low pH; because of this, there is a color change based on pH, which can be used as a pH indicator. Their chemical structure and the resulting color based on pH is shown in Figure 1C. 

Anthocyanins can be further divided based on their substitutions at the skeleton, especially at ring B, or the glycosylation at ring A and C (Figure 1B). Without a sugar attached, they are called anthocyanidins, the most common ones being pelargonidin, cyanidin, peonidin, delphinidin and malvidin [11]. Main sugar moieties are the monosaccharides glucose, arabinose, galactose as well as the disaccharide rutinose (6-*O*-α-l-rhamnosyl-d-glucose) [12]. Generally, Cyanidin is the most abundant anthocyanidin and Cyanidin-3-glucoside the most common anthocyanin [13,14]. 

A further description of the chemistry of anthocyanins can be found e.g., at Mattioli et al. (2020) [15].

Natural dietary sources of anthocyanins are dark berries such as bilberries, blackberries or blackcurrants and red berries such as strawberries, cranberries or redcurrants [13,16,17,18]. Other common fruit sources are cherries, pomegranate and red grapes. In vegetables, they can be found in beetroot, purple corn, red cabbage, black carrots or eggplant [13]. In dark berries, a common and frequently consumed source of anthocyanins, the concentration ranges from around 80 up to 700 mg/100 g fresh weight [13,16,17]. However, these levels vary depending on growth conditions and strain [19,20]. The anthocyanin composition also varies between plants and species. Blackcurrants, for example, comprise of four different anthocyanins (glucoside and rutinoside of cyanidin and delphinidin), whilst bilberries comprise of 15 different anthocyanins (arabinoside, galactoside and glucoside of cyanidin, delphinidin, malvidin, peonidin and petunidin) [14]. Table 1 shows the anthocyanin content in common fruits and vegetables [13,16,17,21,22,23,24,25,26,27,28,29,30,31].

## 3. Absorption, Distribution, Metabolism, Excretion (ADME)

Since anthocyanins, in fruits, vegetables or supplements, are taken up orally, they encounter several different chemical and biological milieus until they are absorbed or excreted and are chemically or enzymatically metabolized. The absorption pathway of anthocyanins seems to deviate compared to the pathway of other flavonoids [32,33]. McGhie and Walton [11] stated a comparatively low bioavailability since only 0.1% of oral cyanidin-3-*O*-glucoside could be found in urine. HPLC as a detection method is debatable since its low pH leads to cation formation in anthocyanins. Manach et al. reviewed studies using anthocyanin amounts ranging from 150 mg to 2 g. Blood plasma concentration of just 10–50 nM were found and retrieval from urine differed from 0.004% to 0.1% [33].

Czank et al. (2013) as well as de Ferrars et al. (2014) used 500 mg ^13^C isotopically marked cyanidin-3-*O*-glucoside (C3G) and collected biological samples over 48 h [34,35]. Mean percentage of recovered ^13^C in urine, breath and feces was 43.9 ± 25.9%. Relative bioavailability was 12.38 ± 1.38% (5.37 ± 0.67% excreted in urine and 6.91 ± 1.59% in breath). They also found a total of 24 labeled metabolites. These included phase II conjugates (cyanidin-glucuronide, methyl cyanidin-glucuronide, and methyl C3G-glucuronide), but also degradants (proto catechuic acid and its own phase II conjugates (i.e., vanillic acid or isovanillic acid), and phloroglucinaldehyde), hippuric acid, and ferulic acid. While C3G serum concentration peaked around 2 h post-consumption, its degradants peaked approx. 6 h post-consumption. Other metabolites, such as vanillic acid or ferulic acid, reached their maximum concentration 11–16 h post-consumption. They were the first to show that the observed effects of anthocyanins in humans may not only be accomplished by anthocyanins themselves, but also by their metabolites [34,35].

Ackermann (2010) studied the effects of several body fluid models on anthocyanins [36]. He found no influence of salivary juice on anthocyanin composition as well as no influence of oral enzymes or bacteria even at longer incubation times. However, he annotates that oral pH is difficult to simulate since it is dependent on food uptake. In a gastric juice model (pH 1.81), containing pepsin, he also found no metabolization over an incubation time of 4 h. This has also been found by others [37,38,39]. However, more recent studies suggested that there might be changes in anthocyanin composition and content in the stomach depending on the type of anthocyanin (i.e., cyanidin or delphinidin) [40]. 

As anthocyanins can be detected in blood serum rapidly after ingestion, it was found that they are absorbed from the stomach without prior metabolization [41,42,43]. Passamonti et al. [41] suggested the bilitranslocase to be involved in this transport. In a human gastric epithelial cell model (NCI-N87), used for studying drug metabolism [44], a non-linear transport of anthocyanins had been found, suggesting an active transport [45]. Similarly, Oliveira et al. found a trans-epithelial transport in a different gastric model (MNK-28). In a computational model, they found anthocyanins to show affinity for the human glucose transporter 1 (GLUT1) [46]. In a further study, silencing of GLUT1 and GLUT3 mRNA resulted in a transport reduction of Malvidin-3-glucoside and other anthocyanins from a purple sweet potato peel extract, suggesting these two transporters to play an important role in anthocyanin transport in the stomach. However, the transporters seem to have different affinities to anthocyanins depending on the glycosylation level [47].

In artificial duodenal juice (pH 7.2), containing pankreatin, a commercial mixture of amylase, lipase, and protease, Ackermann (2010) found a decrease of 8 out of 12 anthocyanins 30 min after incubation [36]. Only the concentration of the Petunidin glycosides and Cyanidin-arabinoside did not decrease. After 10 h of incubation, all anthocyanin concentrations were 12% of base level or below. After 24 h, no traces of anthocyanins could be found. In control solutions, containing no enzymes, he found a similar result, suggesting only an influence of pH rather than enzymes. In ex vivo studies of the small intestine, he found a half-life period of 1.9–4.6 h with delphinidin-glycosides being much more unstable than malvidin- or petunidin-glycosides. In a large intestine model, 1 h incubation resulted in an anthocyanin decrease of up to 90%. It should be noted that the model was composed of porcine caecum instead of human. These results, namely the absorption in the small and large intestine, were also confirmed by others [48,49]. However, the small intestine seems to be the main absorption site together with the stomach (see also [32]).

Additional to these findings, the influence of the gut microflora was also examined. The general influence of small and large intestine microflora on anthocyanin metabolization was discovered early [50,51]. The metabolization products also seem to depend on anthocyanin structure and glycosylation [49,52].

Cassidy and Minihane (2016) reviewed several studies concerning metabolization of flavonoids. In the small intestine, the lactase phlorizin hydrolase (LPH) is considered to hydrolyze glycosides into their aglycones, which then can enter the cells via passive diffusion. However, anthocyanins are an exception to this since they are mainly present as glycosides. Glycosides can enter epithelial cells via transporters like the sodium-dependent glucose transporter 1 (SGLT1) and be hydrolyzed in the cells [47,53]. After absorption, they undergo phase I metabolism in the liver by P450 monooxygenases and phase II metabolism by enzymes like urine-5′-diphosphate glucuronosyltransferases (UGT) or catechol-*O*-methyltransferases (COMT) [54]. In addition, enterohepatic recycling has been found to account, to a degree, for the persistence of anthocyanin conjugates and complexity of metabolites produced from a dose of anthocyanins, thus demonstrating the complexities involved in understanding the bioavailability of anthocyanins [55]. 

**Figure 2:**
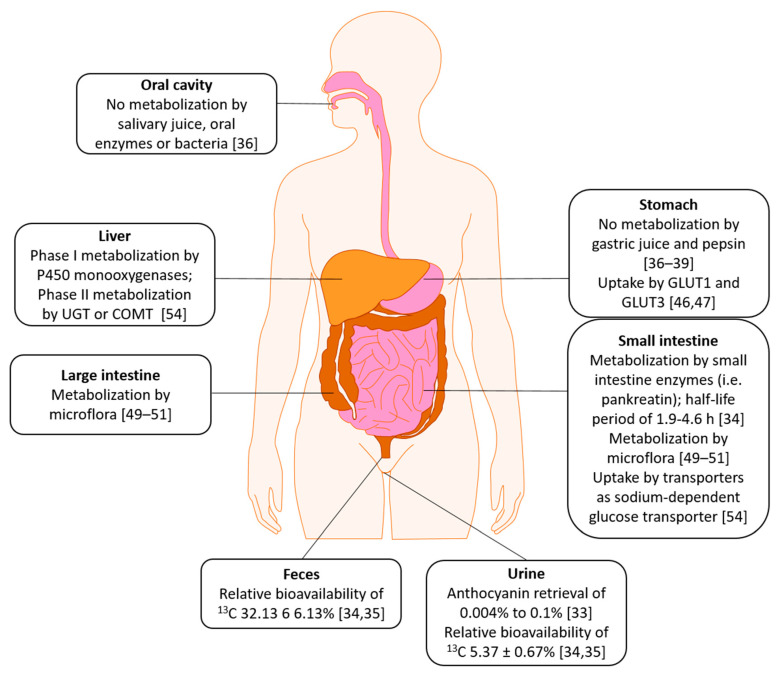
Absorption, Distribution, Metabolism, Excretion of anthocyanins in human; UGT: Uridine 5′-diphospho-glucuronosyltransferase, COMT: Catechol-*O*-methyltransferase; GLUT1: glucose transporter 1; GLUT3: glucose transporter 3.

Overall, anthocyanins seem to have a higher bioavailability than initially thought. They seem to be not influenced by gastric acid but are converted into a variety of metabolites, mainly in the small intestine by enzymes and gut microflora. An overview of the ADME is shown in Figure 2.

## 4. Inflammation

An inflammation is a body’s reaction to a damaging stimulus. This stimulus can be one of three types: Physical, i.e., radiation, temperature, pressure or an injury; chemical, i.e., allergen, toxin or an acid/base, or it can be a biological stimulus, i.e., viral, bacterial or fungal. 

Inflammation includes, but is not limited to, an increase in endothelial permeability, so that extravasation of proteins and signal molecules from the blood stream into surrounding tissue is promoted [56]. In the case of an acute inflammation, this behavior is desirable, but in the case of a chronic low-grade inflammation it can lead to several diseases such as atherosclerosis, where the inside of arteries become narrowed due to plaque formation. More specifically, sub-endothelial accumulation of fatty substances, also called atheroma, occurs [57]. Concerning cardiovascular health, this is not only detrimental because an atheroma can ulcerate, or break, leading to blood clotting, but also because the vessel is constricted. Since the blood flow itself remains nearly the same, the velocity increases, thus leading to further damage [58]. 

In the early stages, important mediators for inflammation within blood vessels are cytokines such as tumor necrosis factor α (TNF-α) or interleukins, as well as prostaglandins [59]. In the second stage, there is an increase in adhesion proteins like monocyte adhesion molecule-1 (ICAM-1), vascular cell adhesion molecule-1 (VCAM-1), or monocyte chemotactic protein-1 (MCP-1) [59]. The transcription factor NF-kB plays a central role in inflammation, activating genes to stimulate the expression of pro-inflammatory markers [60]. 

Effects of single anthocyanins on these markers have been studied in vitro. A model, often used for cell culture studies on the human endothelium, and, more recently, on mechanisms in CVD development, are human umbilical vein endothelial cells (HUVEC) [61]. Huang et al. (2014a) found that with the HUVEC model, it could be demonstrated that both malvidin-3-glucoside (M3G) and -galactoside (M3Gal) had a marked inhibitory effect on the TNF-α-induced increase of ICAM-1 and VCAM-1 protein and mRNA levels in a concentration-dependent manner. When comparing the single anthocyanins directly, M3G exhibited a stronger effect relative to the M3Gal. A combination of both, however, resulted in a synergistic, additive effect. At a combined concentration of 10 μM, the mixture inhibited 98% increased ICAM-1, and 70.1% increased VCAM-1 protein levels in the supernatant [62]. The single anthocyanins only reached this level at concentrations of 50 µM and 100 µM, respectively. Further investigations also demonstrated that the effect of the aglycone malvidin on ICAM-1 and VCAM-1 produced a similar inhibitory effect [63]. The same effect on VCAM-1 was found by Warner et al. (2017), where C3G metabolites (as previously identified in blood plasma by Czank et al. (2013) at different time points in HUVEC, as well as in human coronary artery endothelial cells (HCAEC), suppressed VCAM-1 expression [64]. This suggests an effect not only of anthocyanins itself but also of their metabolites—understanding the activity of anthocyanin metabolites and degradants is a crucial area in order to understand the true scope of systemic impact that anthocyanins may have. In HUVEC, Warner et al. (2017) found a decrease of interleukin 6 (IL-6) by 36.63%, 31.26% and 35.56% for a metabolite mixture reflecting serum concentrations 1, 6 and 24 h after ingestion of 500 mg C3G at cumulative concentrations of 2, 20, and 44 μM [64]. Bharat et al. studied the effect of M3G, C3G plus several of their metabolites on lipotoxicity in HAEC when simulated by palmitate. As palmitate is the most abundant fatty acid in the human body, this model is used to study the effects of excess lipid accumulation. In the human body, this physiological scenario can lead to endothelial dysfunction [65]. When HAEC were exposed to the series of anthocyanins and respective metabolites, they found a reduction of ICAM-1 and VCAM-1 mRNA by the anthocyanins, with a reduction in IL-6 mRNA by the metabolites [66]. Palmitate-induced inflammation was also studied by Muscara et al. (2019) in murine adipocytes [67]. They found that anthocyanins specifically from bilberry (*Vaccinium myrtillus*) and blackcurrant (*Ribes nigrum*) had a dose-dependent attenuating effect on NF-kB and IL-6 mRNA level. This effect was only found after the cells were stimulated with palmitate but not in the control. Krga et al. (2016) used an in vitro model with circulating fluid. They also used anthocyanin and metabolite concentrations found in blood plasma 1–5 h and 15 h after ingestion. While they found a reduction of monocyte-adhesion with C3-galactoside, C3-arabinoside, petunidin-3-glucoside (Pn3G), delphinidin-3-glucoside (D3G), ferulic acid, hippuric acid, and proto catechuic acid, they found no effect on VCAM-1 or ICAM-1 mRNA expression [68]. C3G was also found to attenuate TNF-α induced inflammatory marker increase in HUVEC and rat smooth muscle cells [69,70,71], as well as a lipopolysaccharide (LPS) induced increase [72,73]. Similar results were found for M3G in bovine endothelial cells [74]. 

Anthocyanins have also been studied in vivo. Historically, one approach to this was to use fruit juice in clinical studies. Whilst juice is likely more easily available at lower cost than anthocyanin extracts in capsules, it is difficult to establish a proper control placebo. One example of a juice-based intervention included patients with at least one risk factor for CVD, where they were then asked to consume 330 mL bilberry juice per day for 4 weeks, with a resulting significant decrease in IL-6, IL-15, and C-reactive protein (CRP) reported by Karlsen et al. (2010). Similarly, daily consumption of 240 mL tart cherry juice for 4 weeks was associated with a reduced erythrocyte sedimentation rate, a chronic inflammation marker, in obese patients [75]. In a further clinical trial of healthy adults with an intake of 300 mg anthocyanins extracted from bilberry and blackcurrant daily for 3 weeks, Karlsen et al. (2007) found no difference in plasma lipids between the two groups [60]. However, they found a significant decrease of interleukin 8 (IL-8) by 45% and “regulated upon activation, normal T-cell expressed and secreted” (RANTES) by 40% in the anthocyanin group. Moreover, a non-significant but trending decrease in interleukin 4 (IL-4) and interleukin 13 (IL-13) was recorded. Additionally they found a suppression of NF-kB activation in monocyte cell culture induced by LPS in vitro of 27.6%, although anthocyanin concentration was highly supraphysiological [60]. Zhu et al. also found a decrease in inflammation markers in hypercholesterolemic subjects. They found a significant decrease in human serum C-reactive protein (hsCRP, 21.6%), serum VCAM-1 (12.3%) and interleukin 1β (IL-1β, 12.8%) after 24 weeks intake of 320 mg anthocyanins from bilberry and blackcurrant daily [76]. Hassellund, et al. [77]) found an increase in von-Willebrandt-factor in pre-hypertensive men, which is presumed to indicate adverse changes of the endothelium, after 4 weeks of 640 mg anthocyanins daily. Moreover, interleukin 6 (IL-6) trended towards a significant decrease. However, they found no effect on other interleukins [77]. This result, however, differs from others in the literature. The reasons for that might be the shorter intervention time period and the subjects being pre-hypertensive instead of having metabolic syndrome or dyslipidemia as in other studies [76,78,79,80]. An interleukin-decreasing effect might be only found in this specific subgroup of patients. In this context, Zhang et al. [79] found a significant decrease in serum IL-6 levels over the course of 12 weeks (with an additional significant difference in the mean change compared to the placebo group) in patients with dyslipidemia after daily supplementation of either 40, 80 or 320 mg of anthocyanins sourced from bilberry and blackcurrant [79]. They also found a significant decrease over time of serum TNF-α; no significant changes of note were recorded for the groups receiving either the placebo or 40 mg anthocyanin dose [79]. These effects on inflammation were dose-dependent, with 80 mg/day being the threshold to significance for certain markers. Furthermore, they found the effects to be stronger after 12 weeks than after 6 weeks, although not being linear, suggesting a minimal intervention time [79]. Aboonabi and Aboonabi [78] found a similar result—they recorded a significant downregulation of TNF-α, IL-6 and IL-1A gene expression after supplementing 320 mg anthocyanins for 4 weeks in subjects with metabolic syndrome [78]. In human immune cells, they found a significant increase in peroxisome proliferator-activated receptor gamma (PPAR-γ) mRNA expression. This receptor attenuates inflammatory response by antagonizing proinflammatory transcription pathways, i.e., NFκB [81] and so these intriguing interactions described by Aboonabi and Aboonabi [78] may explain and provide the most clarity so far in terms of the proposed inflammation-attenuating effects of anthocyanins. 

**Figure 3:**
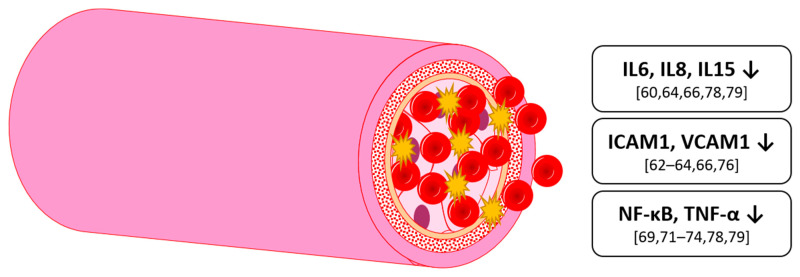
Effects on inflammation markers by anthocyanins in humans and animals; IL: interleukin, ICAM1: intercellular adhesion molecule 1, VCAM1: vascular cell adhesion protein 1, NF-κB: nuclear factor ‘kappa-light-chain-enhancer’ of activated B-cells.

Overall, single anthocyanins exhibited an inhibition of inflammation markers like ICAM-1, VCAM-1 and IL-6 both in vitro and in vivo. Bilberry juice, being rich in anthocyanins, has been shown to decrease IL-6 and IL-15 levels, as well as CRP. Single anthocyanins (M3G and C3G) have also been shown to decrease several interleukins in vivo (Figure 3). These effects appear to be dose- and time-dependent. For significant health benefits, at least in dyslipidemic subjects or other population groups with risk factors for CVD, a minimum amount of 80 mg anthocyanins per day may be beneficial, preferably from dark berries (i.e., bilberry or blackcurrant) as they were used in the studies. This being achievable by eating the equivalent to around two handfuls of dark berries per day [79]. 

## 5. Antioxidative Effects

Oxidation is a chemical reaction, in which a molecule loses an electron, which increases its oxidative state. Compounds that oxidize other molecules are called oxidizers or oxidants. Oxygen, although necessary for complex life, can form several potential harmful oxidants called reactive oxygen species (ROS). The most common examples for these are hydrogen peroxide (H_2_O_2_), the hydroxyl radical (OH) and the superoxide anion (O_2_^−^). They are not necessarily detrimental since they are a natural byproduct of oxygen metabolism and are needed in cell signaling and homeostasis [82]. However, in higher amounts, ROS can damage cellular components, such as DNA, protein or lipids. Thus, an organism has to ensure to maintain an optimal ROS level. This is done by antioxidants which are divided into water-soluble (hydrophilic) and fat-soluble (lipophilic) compounds [83] depending on their location in the cytosol or in cell membranes. In humans, some antioxidants, like ascorbic acid, have to be obtained via diet; others, such as uric acid, can be enzymatically produced. A surplus of ROS, either via overproduction of ROS or via a decrease in antioxidant activity, is called oxidative stress. This can occur either by endogenous sources, such as inflammation mental stress, or ageing, but also by exogenous sources, such as environmental pollutants, cigarette smoke, alcohol or diet (i.e., fatty and smoked meat) [84]. Oxidative stress is a critical component in the oxidation of low-density lipoprotein (LDL) which can lead to diseases like the acute thrombotic event (i.e., heart attack or stroke). In the central nervous system (CNS), oxidative stress has been associated with the so-called neuroinflammation and neurodegenerative diseases such as Alzheimer’s disease [8]. In addition to antioxidant molecules, there are also enzymes capable of an antioxidant function. The most common ones are superoxide dismutase (SOD), catalyzing the disproportionation of superoxide, catalase, catalyzing hydrogen peroxide to water and oxygen, and glutathione peroxidase, reducing lipid hydroperoxides. Besides leakage from mitochondria, where they are produced at oxidative phosphorylation, ROS can be produced enzymatically. The most common enzymes are xanthine oxidase (XO), NADPH oxidase (NOX) and cytochrome P450 [12,85,86,87]. In the case of vascular health, it should be pointed out, that ROS, in combination with NO can form reactive nitrogen species (RNS), making NO less bioavailable and by this, acting as vasoconstrictive [12].

Ascorbic acid and other vitamins have shown antioxidant activity in vitro and in vivo. Polyphenols, however, have shown an antioxidant activity in vitro, which does not automatically mean that they are antioxidant in vivo. The catechol group is thought to be responsible for the antioxidative effect [88] but may undergo metabolization into smaller molecules such as protocatechuic acid after ingestion, thus losing its electron acceptor capability.

Anthocyanins and their metabolites have been extensively studied with regard to their antioxidant activity [12]. In 2015, Edwards et al. studied C3G and its metabolites in human umbilical cord endothelial cells (HUVEC). They found a decrease in angiotensin II-stimulated superoxide production with vanillic acid and proto catechuic acid. Surprisingly, there was an unexpected increase of superoxide with of C3G that was in contrast to the effects of its metabolites [89]. Similarly, Huang, et al. [90] found a significant reduction in ROS level using malvidin-3-glucoside and –galactoside. Further, they found a decrease of xanthine oxidase 1 (XO-1) production. Also, levels of SOD and heme oxygenase 1 (HO-1) were increased, all indicating a high antioxidant potential for these anthocyanins [90]. Lazzè Maria, et al. [91]) also found an increase in HO-1 in HUVEC using the aglycones cyanidin and delphinidin [91]. However, they used concentrations of 50 µM and 100 µM, which are probably supraphysiological. Goszcz, et al. [92]) found a significant decrease in pyrogallol-induced increase of superoxide and SOD by delphinidin and gallic acid [92]. Edwards et al. [89] mention a special role for specific metabolites, most importantly protocatechuic acid and vanillic acid, which resemble the structure of apocynin. The latter being a vasoactive medical drug that specifically inhibits NOX [89].

Anthocyanin-rich foods and pure anthocyanins were also studied in vivo. In healthy subjects, supplementation of 250 mL blackcurrant juice or pomegranate juice daily increased serum paraoxonase/arylesterase 1 (PON1), as well as reduced thiol groups in serum, both markers for serum anti-oxidative status [93]. In a combination of in vivo and in vitro studies, Cimino, et al. [94] gave healthy subjects 160 mg of anthocyanins and took blood plasma samples before and 2 h after ingestion. Human serum Trolox equivalent antioxidant capacity (TEAC) and human serum ferric reducing/antioxidant power (FRAP) were significantly higher after 2 h. They further used the plasma as a 20% additive to cell culture medium and found a protective effect on HUVEC under moderate hypoxic conditions [94]. In a clinical study using 160 mg of anthocyanins from bilberry (*Vaccinium myrtillus*) and blackcurrant (*Ribes nigrum*) daily, in patients with type 2 diabetes for 24 weeks, Li, et al. [95] found the Plasma total radical-trapping antioxidant parameter (TRAP) and Plasma ferric reducing antioxidant power (FRAP) significantly increased and 8-iso-prostaglandin F_2α_ (8-iso-PGF_2α_) decreased [95], TRAP and FRAP being standards for measuring antioxidant properties of blood samples, 8-iso-PGF_2α_ being a biomarker of lipid peroxidation and indicator of oxidative stress [96]. Zhang et al. [79] conducted a similar study by using 40, 80, or 320 mg of anthocyanins, also from bilberry and blackcurrant, daily for 12 weeks in patients with dyslipidemia. They found urine 8-iso-PGF_2α_ being decreased after 12 weeks in the 80 mg and the 320 mg group. 320 mg daily further decreased 8-Hydroxy-2′-deoxyguanosine (8OHdG) and malonaldehyde (MDA), both being markers used for quantifying oxidative stress. In the case of the decreases in 8-iso-PGF_2α_, 8OHdG, and MDA, the effect was dose-dependent (*p* < 0.05).

**Figure 4:**
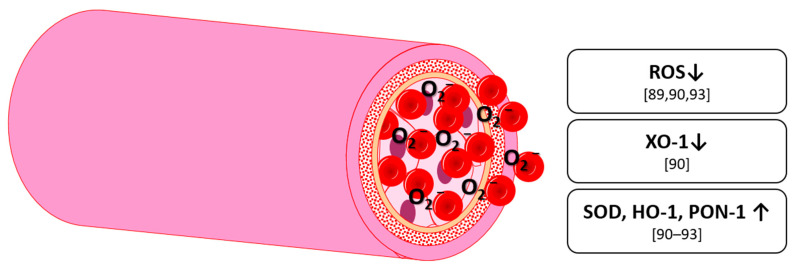
Effects on oxidative stress markers by anthocyanins in humans and animals; ROS: reactive oxygen species, XO-1: xanthine oxidase 1, SOD: superoxide dismutase, HO-1: heme oxygenase 1, PON-1: Serum paraoxonase and arylesterase 1.

Overall, single anthocyanins show a clear antioxidant activity in vitro by influencing several important enzymes. They show a reduction of oxidants, such as ROS and SO-1 and an increase of antioxidant enzymes, such as SOD and HO-1. So far, in vivo studies seem to confirm the in vitro data (Figure 4) and even indicate a potential dose-dependent effect. 

## 6. Vascular Function

For a healthy cardiovascular system, several conditions need to be fulfilled. First, blood vessels need a high level of elastic compliance, so that an efficient propagation of the blood pressure wave along the vessel tree is ensured. Second, on the arteriolar level, endothelial and smooth muscle cells need to function properly to effectively regulate vascular tone and blood flow. This requires an adequate capacity of the endothelial cells to react to mechanical and chemical stimuli and form a sufficient amount of vasoactive substances [97]. Depending on the stimulus, these substances are either called vasodilatory (vessel-widening) or vasoconstrictive (vessel-narrowing).

One of the most studied vasodilators is nitric oxide (NO), which was also known as an endothelium-dependent relaxing factor (EDRF). After synthesis, NO interacts with soluble guanylate cyclase (sGC), resulting in the conversion of guanosine triphosphate (GTP) to cyclic guanosine monophosphate (cGMP) [98]. In endothelial cells, NO is synthesized by the endothelial nitric oxide synthase (eNOS), stimulated by shear stress. eNOS is a dimer, consisting of a reductase domain with binding sites for nicotinamide adenine dinucleotide phosphate (NADPH), flavin mononucleotide (FMN) and flavin adenine dinucleotide (FAD) and an oxidase domain with binding sites for heme group, zinc, tetrahydrobiopterin (BH4) and the substrate l-arginine [99]. Stoichiometry of the reaction is as follows:

2 l-arginine + 3 NADPH + 4 O_2_ → 2 l-citrulline + 4 H_2_O + 2 NO

The effect of anthocyanins on vasodilation and especially eNOS has been extensively studied. In an in vitro study in HUVEC, Edwards et al. found an upregulation of eNOS expression up to 700% of the control value when they incubated the cells with C3G, while there was no effect with proto catechuic acid or vanillic acid [89]. Edirisinghe, et al. [100]) used glucosides and rutinosides of cyanidin and delphinidin in HUVEC and found a significant upregulation of eNOS, more precisely its phosphorylated form, for each anthocyanin. In a mixture of all four, they also found an upregulation in eNOS. However, there was no additive effect of the components [100]. Another study showed the influence of strawberry, wild blueberry and cranberry extract on PI3 kinase/protein kinase B (Akt), a kinase belonging to an eNOS activation pathway [101]. An upregulation of eNOS has also been found with the anthocyanidins cyanidin and delphinidin [91]. However, the concentrations used were, again, highly supraphysiological. They also found a decrease in the vasoconstrictor endothelin-1 (ET-1). 

There is also evidence of anthocyanin influence on the renin-angiotensin-aldosterone system. Angiotensin is a peptide hormone causing vasoconstriction and increasing blood pressure by acting on venous and arterial smooth muscle cells. The precursor angiotensin I is converted into the vasoactive form angiotensin II by the angiotensin-converting enzyme (ACE) [102]. ACE-inhibitors are a common treatment in patients with hypertension, although they can have several severe side effects, going as far as resulting in angioedema [103]. In 2001, Lacaille-Dubois et al. found the first evidence for ACE inhibition with procyanidins [104]. An in vitro study by Persson, et al. [105] found a reduction in ACE-activity, measured via cleavage of ^3^H-hippuryl-glycyl-glycine, with a blueberry extract as well as with delphinidin-3-glucoside. They found no effect with the aglycones and suggest the missing glycosylation as a cause [105]. Hidalgo, et al. [106] studied the effect of several anthocyanins on ACE-inhibition by determining the IC_50_ value. This value is the concentration needed to inhibit enzyme activity down to 50% of its original activity. They found D3G and C3G to have the lowest IC_50_ with Pel3G, M3G and Peo3G, as well as metabolites like gallic acid or coumaric acid to have up to five times higher values [106]. A similar has been found with delphinidin- and cyanidin-3-O-sambubiosides from *Hibiscus sabdariffa* [107]. This could show an ACE-inhibiting effect of specific anthocyanins only, while other anthocyanins and metabolites have no effect.

Measuring of vascular function in vivo is achieved by measurements of blood pressure (BP), pulse wave velocity (PWV), or flow mediated dilation (FMD). For PWV there are two common measurements: first is the carotid-femoral PWV (cfPWV), second is the brachial-ankle PWV (baPWV), both measuring at different points but aiming to capture the speed of the waveform as it progresses along the arterial tree [108]. For FMD, the dilation of the brachial artery is measured via ultrasound after the application of artificial forearm ischemia (e.g., via a cuff) and subsequent release. 

In an ex vivo study on coronary rings, Bell and Gochenaur [109]) found a relaxation when treated with a chokeberry or a bilberry extract. This effect was diminished with either removal of endothelium or addition of a NO-synthase inhibitor. Similarly, Kalea, et al. [110] found a diminished vasoconstrictor response in aortic ring from blueberry-fed rats. This endothelium-dependent effect was also found by others [111,112,113,114]. When studied in vivo, rats fed a blueberry extract showed a significantly decreased systolic blood pressure [115], which is in line with the ex vivo studies.

Khan, et al. [116] found a significant increase in FMD after supplementation of 1 L blackcurrant juice per day for 6 weeks in healthy subjects. Similar results were found by Rodriguez-Mateos, et al. [117] with intake of blueberry polyphenols, containing approx. 1/3 anthocyanins. They also found the FMD increase being biphasic time dependent. These peaks were accompanied by a biphasic increase of plasma polyphenol metabolites, with vanillic acid and benzoic acid levels predicting FMD increase at 1–2 h post-consumption and hippuric acid, homovanillic acid and hydroxyhippuric acid predicting the peak at 6 h [117]. Anthocyanin-rich tart cherry juice concentrate failed to show an effect on arterial stiffness and blood pressure in healthy patients [118], but there was an effect seen in hypertensive patients [119]. Furthermore, it has been recorded that an infusion of anthocyanins resulted in an improvement of FMD by 28.4% (that was subsequently blocked by an infusion of monomethyl-l-arginine-acetate, a NOS-inhibitor) and a concomitant 12.6% increase of plasma cGMP level [120]. The latter correlating positively with FMD, suggesting involvement or recruitment of the NO-cGMP signaling pathway [120]. In post-menopausal women in the pre-hypertensive stage, an intake of 22 g freeze-dried blueberries daily for 8 weeks, significantly decreased systolic and diastolic BP by 5.1% and 6.3% respectively, as well as baPWV by ~7% [121]. The same effect was found with intake of cranberry juice [122]. In adipose patients with metabolic syndrome, Basu, et al. [123] found significantly lowered systolic and diastolic blood pressure after intake of 50 g freeze-dried blueberries daily by 6% and 4% respectively. However, this was not a double-blind study as the control drink was pure water, which might have had an influence on the outcome. Curtis, et al. [124] found a total increase of 1.45% in FMD and a 2.24% decreased augmentation index after supplementation of 364 mg anthocyanins from freeze-dried blueberry powder, equivalent to 1 cup or 150 g fresh blueberry, in patients with metabolic syndrome after 6 months [124], and therefore improved endothelial function and reduced stiffness. In another study, supplementation of 320 mg anthocyanins from bilberry and blackcurrant over 12 weeks resulted in increased FMD and adiponectin in type 2 diabetic patients [114]. 

In a set of experiments in healthy males, Rodriguez-Mateos, et al. [125] found that consumption of a blueberry drink containing 150 mg of anthocyanins or pure anthocyanins as well as fiber and minerals, both increased FMD in a similar magnitude. Furthermore, a dose-dependent effect on FMD at 2 and 6 h post-consumption was found with 0–480 mg of anthocyanin. They then administered 300 mg of anthocyanins from wild blueberry powder per day for 4 weeks. They found an increased FMD after one week, with a plateau after 2 weeks, suggesting at least 2 weeks of consumption to achieve a sustained improvement. In a fourth setup, using the same design, FMD was increased by 2.3% after 4 weeks. However, acute consumption on the last day did not show further FMD improvement. This suggests an effect saturation as well as that acute and long-term effects might be mediated by similar pathways [125]. An additional metabolomics analysis revealed fourteen phenolic metabolites to significantly correlate with the acute effects and 21 with the chronic responses, with nine of them correlating with both acute and chronic responses [125]. 

However, supplementation of 640 mg bilberry (*Vaccinium myrtillus*) and blackcurrant (*Ribes nigrum*) anthocyanins over 4 weeks did not result in significant changes in blood pressure in healthy men [126]. This was also confirmed by others [127,128,129], suggesting no, or only minor effect in healthy humans.

**Figure 5:**
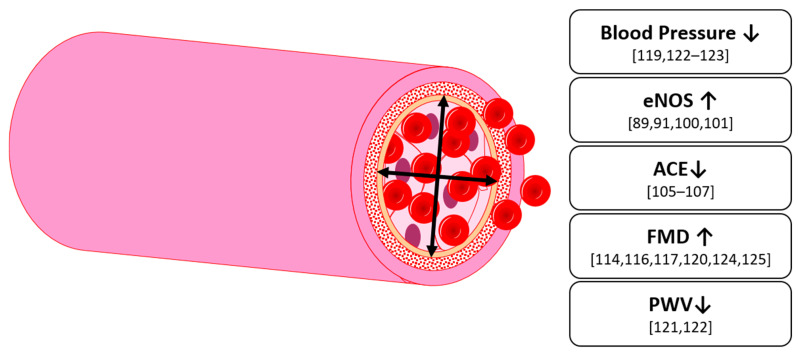
Effects on vascular function by anthocyanins in humans and animals; eNOS: endothelial nitric oxide synthase, ACE: angiotensin converting enzyme, FMD: flow mediated dilation, PWV: pulse wave velocity.

Overall, anthocyanins have been shown to upregulate eNOS in vitro, which is responsible for the synthesis of the vasodilatory NO. Correspondingly, an improved endothelium-dependent vasodilation was found in vivo (Figure 5). There is also evidence of an effect as an ACE-inhibitor in vitro, which would result in a lowered blood pressure. These effects, however, seem not to occur in healthy patients, but patients with hypertension, hyperlipidemia or metabolic syndrome.

## 7. Hyperlipidemia

Abnormally elevated levels of lipids, cholesterol or lipoproteins are well-documented risk factors for cardiovascular disease (CVD) [130]. Although being modifiable risk factors, since they can be improved to a certain degree by lifestyle changes (e.g., changes in diet, weight loss and increased exercise) or drugs, (e.g., statins), a poor lipid profile very likely contributes to the new cases of CVD in Europe each year, such as the recorded 19.9 million cases in 2017 alone [131]. 

Atherosclerosis is a chronic inflammatory vascular disease that is, among other factors mentioned above, associated with hyperlipidemia [132]. In the case of either an increased uptake of LDL cholesterol or a reduced amount of high-density lipoprotein (HDL) cholesterol, oxidized LDL can be taken up by endothelial cells and later macrophages [133]. An increase in HDL in patients is a favorable outcome, due to the likely resulting increase in capacity for reverse cholesterol transport from the arterial plaques to the liver [134]. 

Oxidized LDL can also be used in vitro to induce an endothelial dysfunction model for atherosclerosis [135]. D3G, C3G, but also their metabolites, e.g., protocatchuic and vanillic acid, were shown to have an attenuating effect on these damaging effects in vitro [136,137,138,139]. In mice, anthocyanin-rich extracts from black rice, decreased the total cholesterol level by 62% and triglyceride level by 54% [140] and mulberry extract decreased cholesterol level by 22% [141]. 

The effect of anthocyanins on hyperlipidemia has been examined in several human studies. In studies conducted with healthy subjects, no significant change in LDL was found using pure anthocyanins or anthocyanin-rich extracts [127,128,129,142,143]. The same applies to triglyceride levels [60,127,128,129,142,143]. One clinical trial found an increase in HDL-C in healthy subjects [128]. In former smokers, however, consumption of 500 mg aronia extract, containing 45.1 mg anthocyanins, for 12 weeks resulted in a decrease in LDL-C [144]. In obese men and women with metabolic syndrome, 50 g of freeze-dried blueberries per day over 8 weeks resulted in a significant decrease of oxLDL by 28% [123]. The same effect was found with cranberry juice in obese women [145]. In slightly overweight subjects (mean body-mass-index (BMI) = 25 kg/m^2^) with type 2 diabetes, a total daily intake of 320 mg anthocyanins from bilberry (*Vaccinium myrtillus*) and blackcurrant (*Ribes nigrum*) for 24 weeks significantly lowered serum low density lipoprotein-cholesterol (S LDL-C) by 7.9% while increasing serum high density lipoprotein-cholesterol (S HDL-C) by 19.4% [96]. They also found a decrease in apolipoprotein B48 and C-III by 16.5% and 11.0%, both being in contact with LDL. Similar results were found by Qin et al. [134]) in slightly overweight subjects (mean BMI = 26 kg/m^2^) in a 12-week clinical trial. They found an increase in HDL cholesterol levels by 13.7% and a decrease in LDL cholesterol by 13.6%. Although they found no difference in total cholesterol or apolipoproteins [134], they also found a decrease in Cholesteryl-ester transfer protein (CETP)-mass and -activity and suggest this to be a possible target of anthocyanins [134]. CETP, a plasma protein, facilitates the transport of cholesteryl-esters and triglycerides between lipoproteins by exchanging them from HDL to LDL, which is then transported to the periphery. It has been shown that inhibition of CETP results in an increase of S HDL-C [146]. These effects on lipoprotein profiles were also found in a study performed with 150 hypercholesterolemic subjects over 12 weeks [120]. A significant increase in HDL cholesterol levels by 12.3% and a decrease in LDL cholesterol by 11.6% was observed. Besides the significant improvement of lipoprotein profiles, flow-mediated dilation (FMD) was also increased [120]. In addition, upon a longer supplementation period of 24 weeks in 150 and 122 hypercholesterolemic subjects, significant increases in HDL cholesterol and decreases in LDL cholesterol were observed. HDL cholesterol increased respectively 14 and 11%. LDL cholesterol decreased respectively by 10.4% and 9.7% [120,147]. Additionally, an increase in HDL-associated esterase/lactonase paraoxonase 1 (HDL-PON1) was found. This Calcium-dependent esterase hydrolyzes oxidized phospholipids and is anti-atherosclerotic. Zhu et al. [120]) also found a significant increase in serum cGMP-concentration by 12.6% as well as an improvement in FMD in the longer-term after 12 weeks, as well as short-term at 1 h post consumption, of 320 mg anthocyanins from bilberry and blackcurrant. In patients with metabolic syndrome, Aboonabi and Aboonabi [78] found, that 320 mg anthocyanins from bilberry and blackcurrant per day, significantly decreased serum TG by 24.9% and decreased LDL-C by 33.1%.

In contrast to these results, Zhang et al. [80]) found no significant changes in overweight subjects (mean BMI = 27) with non-alcoholic fatty liver disease while having the same protocol as Zhuet al. [120]) and Li et al. [95,96]. While there were no changes in total cholesterol or S LDL-C, they found a slight increase in S HDL-C, although not being significant since the same increase was found in the control group [80]. In a 4 week short-term crossover study of 640 mg anthocyanins daily on pre-hypertensive men (blood pressure 140–180/90–110 mmHg), Hassellund, et al. [77] only found a significant increase in S HDL-C while there were no changes in S LDL-C or total cholesterol.

Recently, Xu, et al. [148]) conducted a study, using 0, 40, 80 and 320 mg anthocyanins from bilberry and blackcurrant daily in patients with dyslipidemia. They found a dose-dependent increase in cholesterol efflux capacity after 12 but not after 6 weeks. This is interesting, as Zhang et al. [79] also found a dose-dependent effect with key inflammation markers such as IL6 and TNF-α. 

**Figure 6:**
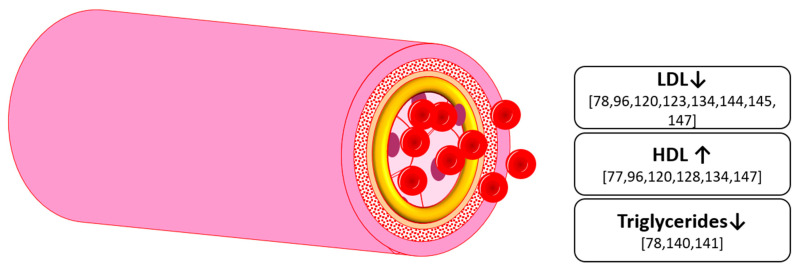
Effects on hyperlipidemia markers by anthocyanins in humans and animals; LDL: low-density lipoprotein, HDL: high-density lipoprotein.

Overall, the intake of up to 320 mg anthocyanins for up to 24 weeks showed a decrease in S LDL-C and an increase in S HDL-C (Figure 6). However, these results were found in subjects with either diabetes, hypercholesterolemia, overweight or pre-hypertension. It is unclear whether a significant effect can be found in healthy subjects. However, this might not be needed, as this group should have lipid levels already in a healthy range. Anthocyanins could then act as a preventative supplement keeping levels in a healthy range. In addition, there seems to be minimal effective dose, as lower doses of 50 mg anthocyanins did not show an effect [149,150] yet a daily dose of 80mg and upwards has been found to be effective in other key aspects of metabolic health, such as inflammation [79]. This effect on the dynamics of the lipid profile then seems to be dose-dependent [148]. 

## 8. Glucose Homeostasis

Epidemiological studies have found an inverse correlation between berry or anthocyanin intake, and the risk of type 2 diabetes (T2D) [151]. Others found anthocyanin intake inversely correlated to insulin level and insulin resistance [152]. 

In a T2D mouse model, black soybean seed-rich extract lowered glucose level and improved insulin sensitivity. This was considered because of activation of the adenosine monophosphate-activated protein kinase (AMPK) in skeletal muscle and liver [153]. In a cross-over study [154], healthy subjects received a berry puree made of bilberries, blackcurrants, cranberries and strawberries, sweetened with 35 g sucrose or a control meal having a similar glycemic profile. Glucose levels 15 and 30 min post-consumption were significantly lower at the berry puree. After 3 h, no difference could be seen [154]. This indicates a delayed digestion of sucrose in presence of berry polyphenols. 

In patients with type 2 diabetes who were also overweight, van’t Erve et al. [96] found a significant decrease in fasting plasma glucose compared to the control group after 24 weeks of a bilberry and blackcurrant anthocyanin supplement (320 mg/day). They also found a decreasing effect in the homeostatic model assessment insulin resistance (HOMA-IR) by 13% [96]. In non-diabetic patients, Zhang et al. [80] also found a significant decrease in HOMA-IR in the anthocyanin group compared to the baseline, but not to the control group, while having the same study protocol as van’t Erve et al. [96]. However, their trial lasted for just 12 weeks. A further 12-week randomized, double-blind, placebo-controlled trial, with a total of 160 Chinese participants aged 40–75 years with prediabetes or early untreated diabetes, was enrolled to investigate the effect of anthocyanins from bilberry and blackcurrant [155]. While there was no difference in fasting insulin, fasting C-peptide, fasting glucose or 2-h glucose between groups post-intervention, they found a slight, but significant decrease in glycated hemoglobin A1C (HbA1c), a biomarker of three-month average plasma glucose concentration [156]. Aboonabi and Aboonabi [78]) found a significant decrease in fasting blood glucose for individuals identified as having metabolic syndrome, after supplementing 320 mg anthocyanins daily for 4 weeks compared to baseline, suggesting an glucose lowering effect at least in higher anthocyanin doses [78]. Finally, Yang et al. also found a decrease in fasting glucose after a 12-week supplementation of anthocyanins from bilberry and blackcurrant (320 mg daily) [157].

**Figure 7:**
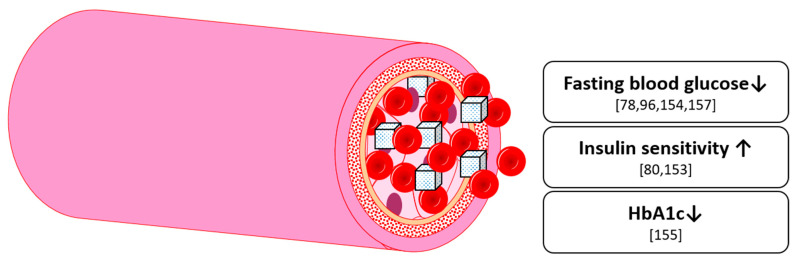
Effects on glucose homeostasis by anthocyanins in humans and animals; HbA1c: Glycated hemoglobin A1c.

Overall, there is a growing volume of evidence on the effect of anthocyanins on glucose homeostasis (Figure 7). However, whilst there seems to be a small yet relevant effect on diabetic subjects from long-term supplementation, an effect in non-diabetic subjects requires further substantiation. Overall, this field is still in an early phase of understanding, and thus quantifying, the role of anthocyanins in glucose metabolism in the human body, therefore further investigation is warranted. 

## 9. Cognition

In several in vitro and in vivo studies, the beneficial effects of flavonoid-rich fruits, such as berries, on neurodegenerative and cognitive outcomes have been found [158,159,160,161]. Several studies have confirmed that the efficacy of anthocyanins in neurodegeneration is primarily due to their anti-oxidative, anti-inflammatory and anti-apoptotic activities, which act against the main hallmarks of neurodegenerative diseases [162,163]. Moreover, specific pathology hallmarks can be improved. For example, it was previously shown in an Alzheimer’s Disease (AD) mouse model that supplementation with anthocyanin-enriched bilberry and blackcurrant extracts lower the deposition of β-amyloid proteins in the brain [164]. Aside from the improvement on a molecular levels, behavioral improvements were also observed. The anthocyanins also improved spatial working memory in a delayed alternation task and a Morris swim task when compared to the control mice [164]. In a different Alzheimer’s mouse model, supplementation of anthocyanins extracted and purified from grape skin improved memory function and prevented anxiety-related behavior [165]. In an in vitro model, transfected with the most used and characterized double-mutation used in AD mouse models (APP Swedish KM670/671NL), the influence of anthocyanins has been studied with respect to mitochondrial function [166]. AD is associated with a decrease in mitochondrial enzymes, especially in complex IV [167], yet there was no effect seen on complex IV post-treatment with anthocyanins. However, in a further study, there was a finding of a significant improvement of rotenone-induced attenuation of complex I, showing a mitochondria-protecting effect via the attenuation of ROS production [168]. The study also described a suppression of the rotenone-induced rise in dynamin-related protein 1 (Drp-1), which is responsible for mitochondrial fission [169]. The impact of oxidative stress in general is a huge risk factor in developing AD, as it can lead to membrane disruption or lipid peroxidation [170] and thus the cellular impact of anthocyanins in an anti-oxidative capacity is important to study. Low antioxidant levels have been linked to AD pathologies [171], therefore an influence of anthocyanins on ROS production could potentially be beneficial in preventing AD.

Another possible contributor to cerebrovascular health is the influence on endothelial cells and consequently, on peripheral blood flow. The positive influence of anthocyanins on blood vessels has previously been described in chapter VI. An efficient cerebral blood flow (CBF) is important, as it is known to facilitate neurogenesis in the hippocampus and there is a positive correlation between CBF and cognitive function in humans [162]. 

Human studies related to anthocyanins and neurodegeneration have presented beneficial effects on behavioral outcomes. In a randomized controlled trial of 12 participants, it was shown that older adults with mild cognitive impairment were able to increase learning and memory capacity after 12 weeks of grape juice supplementation compared to the placebo group [172]. Similar findings were reported after a 12-week supplementation of blueberry juice in the same group, i.e., improved memory function was discovered [173]. 

**Figure 8:**
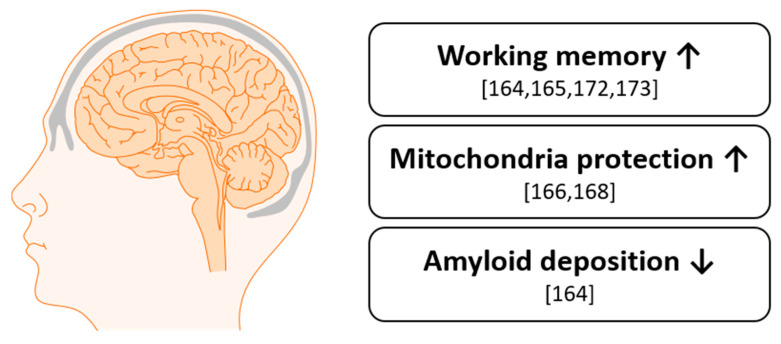
Effects on cognition by anthocyanins in humans and animals.

Overall, anthocyanins appear promising as a possible solution to the growing incidence of neurodegenerative diseases in our aging population (Figure 8). In AD mouse models, cognitive improvements were shown. Nevertheless, further and larger clinical trials in humans studying the effect of anthocyanins on neurodegenerative diseases will have to be pursued. In Western societies, case numbers of diseases like AD are growing [174]. Non-pharmaceutical options, such as supplementation with anthocyanins, might have a future role to prevent or attenuate these diseases. 

## 10. Conclusions

**Figure 9:**
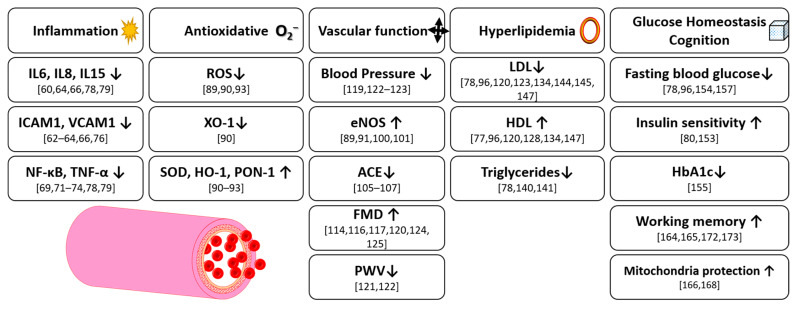
Overview of anthocyanin effects in humans and animals.

Overall, anthocyanins have been recorded as having wide-ranging, quantifiable effects on key CVD risk factors in humans (Figure 9). As has been demonstrated, they significantly reduce inflammation markers such as interleukins and inflammation-related enzymes. Additionally, they show a high antioxidative potential by decreasing reactive oxygen species, as well as upregulating corresponding enzymes. As a consequence, evidence indicates an ability for anthocyanins to directly affect the function of the circulatory system, as evidenced by reducing blood pressure, reducing pulse wave velocity and increasing flow mediated dilation. In hyperlipidemia, anthocyanin supplementation results in a decrease of total cholesterol and LDL, as well as an increase in HDL. There is also evidence of a potential blood glucose lowering effect, as well as an improvement in cognitive health. Therefore, anthocyanins are a promising tool for the prevention of progressive disease states that have a real impact on the health of the population. However, the degree of any effect seems to be dependent on the source of anthocyanins and therefore the spectrum of anthocyanins present. Whilst the breadth and depth of the literature base for anthocyanins is impressive when compared to other plant extracts, as is natural with apparently multi-functional molecules, more clinical trials are needed in humans to confirm promising in vitro or animal study results, particularly with respect to cognitive health. It is also essential that greater understanding is developed in what role single anthocyanins have to play and how their functional capabilities may be changed or enhanced when combined with other anthocyanins. 

A further striking element of anthocyanins is the overall consistency in the effect reported from human trials, particularly for the suppression of inflammation, amelioration of the lipid profile and improved anti-oxidative capacity. With research now also becoming more targeted on understanding effective dose sizes, how anthocyanins are dynamically influencing the transcriptome and pushing to understand the true scope of the effects anthocyanins may have on the body and consequently, health, the field of anthocyanins research is moving into an exciting new phase. 

## Figures and Tables

**Table 1:** Common natural food sources for anthocyanins and their respective anthocyanin content.

Food Source	Anthocyanin Content [mg/100 g Fresh Weight]	Reference
Beetroot (*Beta vulgaris* L.)	23–77	[24]
Bilberry (*Vaccinium myrtillus*)	300–698	[13,17,25,28]
Black carrots (*Daucus carota* ssp. *sativus* var. *atrorubens* Alef.)	1.5–190	[27,30]
Blackberry (*Rubus fruticosus* L.)	83–326	[13,16]
Blackcurrant (*Ribes nigrum*)	130–476	[13,16,17,25]
Blueberry (*Vaccinium angustifolium/corybosum*)	25–495	[13,16,25,28]
Cranberry (*Vaccinium macrocarpon*)	46–200	[13,16,17,25]
Elderberry (*Sambucus nigra* L.)	200–1560	[16,17,29]
Maqui berry (*Aristotelia chilensis*)	137–1250	[22,23]
Pomegranate (*Punica granatum*) juice	9–765 mg/L	[13,21,31]
Purple corn (*Zea mays indurate*)	68–1642	[13,26]
Red cabbage (*Brassica oleracea* L. var. *capitata* L.)	250–322	[13,16,17]
Red Grape (*Vitis vinifera*)	26–750	[13,16,17,25]
Redcurrant (*Ribes rubrum*)	12–22	[13,16,17,25]
Strawberry (*Fragaria* × *ananassa*)	12–55	[13,16,17,25]
Tart cherry (*Prunus cerasus*)	2–450	[13,16,17,25]
